# Risk Factors for Diabetic Peripheral Neuropathy, Peripheral Artery Disease, and Foot Deformity Among the Population With Diabetes in Beijing, China: A Multicenter, Cross-Sectional Study

**DOI:** 10.3389/fendo.2022.824215

**Published:** 2022-06-06

**Authors:** Jiayi Liu, Xiaoyong Yuan, Jin Liu, Geheng Yuan, Yalan Sun, Donghui Zhang, Xin Qi, Huijuan Li, Junqing Zhang, Bing Wen, Xiaohui Guo

**Affiliations:** ^1^ Department of Endocrinology, Peking University First Hospital, Beijing, China; ^2^ Plastic and Burn Surgery Department, Peking University First Hospital, Beijing, China

**Keywords:** peripheral neuropathy, peripheral artery disease, foot deformity, risk factors, diabetes

## Abstract

Diabetic peripheral neuropathy (DPN), peripheral artery disease (PAD), and foot deformity are the most common causes of diabetic foot, which can considerably worsen the patient’s quality of life. In this study, we aimed to investigate the prevalence and risk factors associated with DPN, PAD, and foot deformity among patients with diabetes living in Beijing, China. In total, 3,898 diabetes patients from 11 hospitals in Beijing were evaluated using questionnaires and physical examinations, and 3,758 patients were included in the analysis. We compared the demographic, clinical, biological characteristics, and comorbidities of patients with and without DPN, PAD, or foot deformity, and used binary logistic regression analysis to identify potential factors associated with these outcomes. Overall, 882 patients (23.5%) had DPN, 437 patients (11.6%) had PAD, and 1,117 patients (29.7%) had foot deformities, including callus. The risk factors for DPN included: age ≥40 years, a ≥10+year duration of diabetes, a body mass index of <18.5 kg/m^2^ or ≥24 kg/m^2^, a systolic blood pressure (SBP) of ≥140 mm Hg, a hemoglobin A_1c_ (HbA_1c_) level of ≥7%, chronic kidney disease, and cerebrovascular disease. The risk factors for PAD included: a 15+ year diabetes duration, a body mass index of <18.5 kg/m^2^, a SBP of ≥140 mm Hg, a HbA_1c_ level of ≥7%, chronic kidney disease, coronary heart disease, and cerebrovascular disease. The risk factors for skeletal foot deformities included: women, age ≥40 years, a SBP ≥140 mm Hg, and hyperlipidemia. The risk factors for callus formation included: women, a SBP ≥140 mm Hg, and hyperlipidemia. In conclusion, the prevalence of foot deformities was higher than DPN and PAD in patients with diabetes. Managing the risk factors for DPN, PAD, and foot deformity is important for reducing the risk of diabetic foot.

## Introduction

Diabetic foot is a serious complication of diabetes, associated with a high prevalence and mortality, as well as an enormous medical burden. The annual incidence of diabetic foot is roughly 2%, with a lifetime incidence rate of 15−20% (1). Curing diabetic foot is difficult; even if the ulcer heals, the 1-year recurrence rate is 30–40% (2, 3). The early identification of at-risk patients is therefore crucial.

Intrinsic conditions, including neuropathy, vascular disease, and foot deformity, cause diabetic foot, as do extrinsic risk factors, such as unexpected trauma and infection. The most common extrinsic risk factors for diabetic foot are diabetic peripheral neuropathy (DPN) and peripheral artery disease (PAD) (4, 5). International consensus guidelines define DPN as the presence of symptoms or signs of peripheral nerve dysfunction in patients with diabetes after all other causes have been ruled out (6). The International Working Group on the Diabetic Foot (IWGDF) proposed the concept of graded at-risk foot screenings, which emphasizes the importance of screening for high-risk factors of foot disease among diabetic patients, including DPN, PAD, foot deformity, foot ulcers, and partial foot or leg amputation history (7). Early screening for PAD can reduce the occurrence of diabetic foot (8). In our previous research, through Delphi consultation, we developed the “Construction of the standardized process of at-risk foot screening, stratification and intervention for diabetic patients” (hereafter referred to as the Screening Process criteria), which provides a framework for the comprehensive management of diabetic foot in China (9). Overall, 47.1% of patients with diabetes who met the Screening Process criteria had foot diseases, including DPN, PAD, and foot deformity (10). Therefore, patients with diabetes who meet the Screening Process criteria have a higher risk of developing diabetic foot, and should be given special attention towards preventing this complication.

To the best of our current knowledge, there are limited data on this patient population. This study screened patients with diabetes in multiple outpatient clinics in Beijing, China using the Screening Process. We aimed to estimate the prevalence and risk factors associated with DPN, PAD, and foot deformity among patients with type 1 and 2 diabetes, and potentially enhance preventive measures and care for patients with diabetes.

## Materials and Methods

### Subjects

The sample consisted of 3,898 consecutively enrolled outpatients who underwent a screening for diabetic foot between June 1, 2017 and January 14, 2019 and were diagnosed with diabetes across 11 hospitals located in Beijing, China. Peking University First Hospital, Dongzhimen Hospital Beijing University of Chinese Medicine, Beijing Jishuitan Hospital, Air Force General Hospital of PLA, Peking University Shougang Hospital, Aerospace Center Hospital, Beijing Pinggu Hospital, Beijing Miyun Hospital, Beijing Shijingshan Hospital, Beijing Shichahai Community Service Center, and the Beijing Xinjiekou Community Service Center participated in the study.

**Figure 1:**
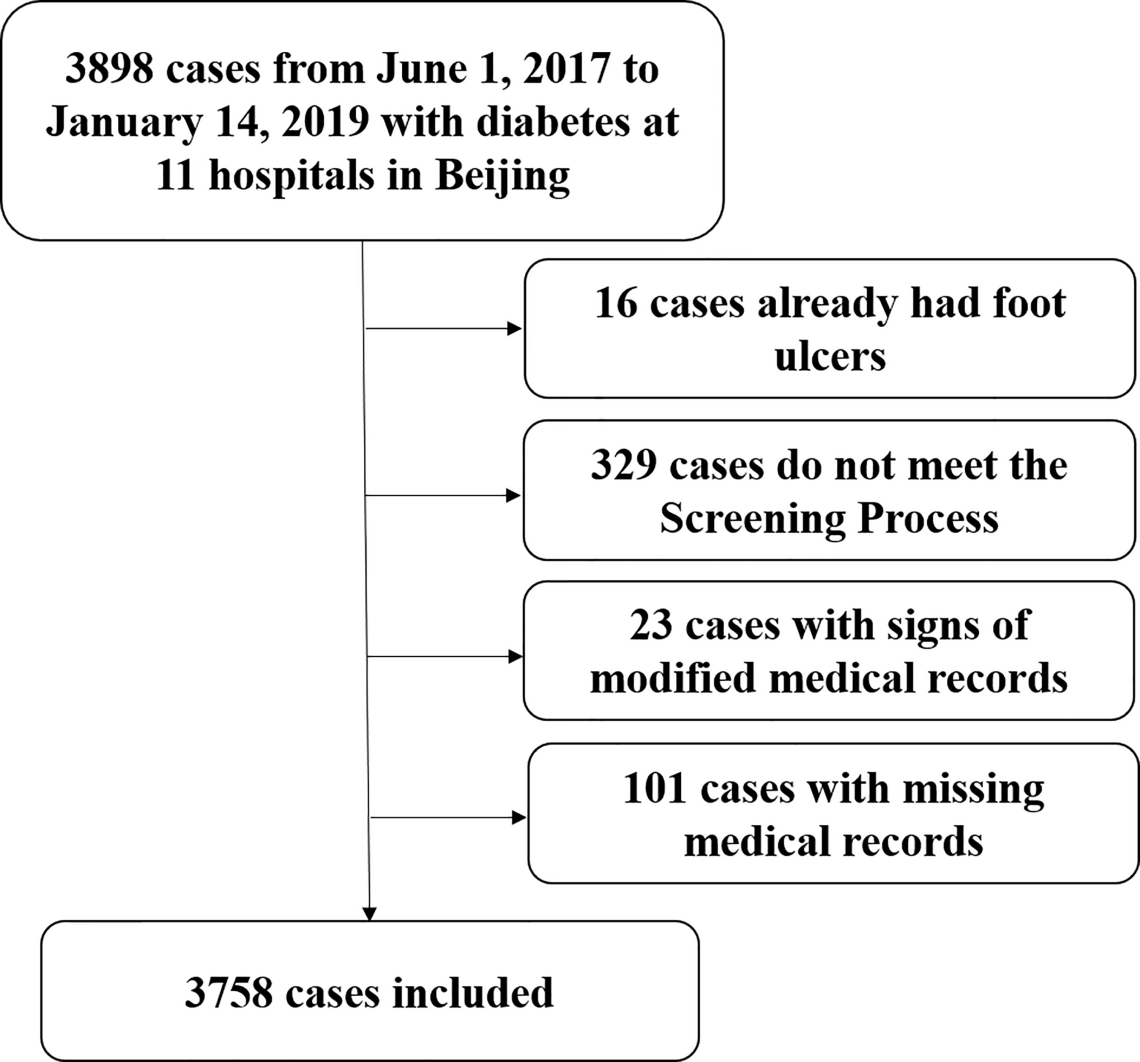
Flow chart of diabetic patient enrollment.

Men and women with type 1 or type 2 diabetes mellitus who were conscious, without language communication barriers, and willing to actively cooperate with the evaluation were eligible to participate. Diabetes was diagnosed based on the 1999 World Health Organization criteria (11). If the patient met any of the following criteria(Screening Process criteria) (9), he or she was included in this study: age > 60 years old; duration of diabetes > 8 years; history of PAD; history of DPN; history of diabetic nephropathy; history of diabetic retinopathy; history of foot deformity, including skeletal deformity of the foot and callus; history of diabetic foot and/or amputation. Exclusion criteria included: patients with existing foot ulcers, and patients whose medical records were missing relevant data (e.g., foot examination or the ankle-brachial index [ABI] results) (**Figure 1**).

In total, 3,758 patients with diabetes were included in the final sample. The institutional review board at each study site approved this study, and informed consent was obtained from all participants before the survey was conducted.

### Data Collection

Prior to study initiation, all doctors and nurses at each site were adequately trained in operations manual. Data on sex, age, diabetes duration, and accompanying disease history (e.g., hypertension, hyperlipidemia, chronic kidney disease, coronary heart disease, and cerebrovascular disease) were collected upon admission. Systolic blood pressure (SBP) and height and weight (excluding shoes and heavy clothes) were also measured on admission. Body mass index (BMI) was calculated as the weight in kilograms (kg) divided by the height in meters squared (m^2^). The most recent hemoglobin A_1c_ (HbA_1c_) and low-density lipoprotein cholesterol (LDL-c) levels were verified. HbA_1c_ and LDL-c are uniformly tested by the central laboratory. The symptoms of DPN and PAD, including rest pain, intermittent claudication, numbness, and paresthesia, were collected by trained nurses. Physical examinations to diagnose DPN, PAD, or foot deformity were conducted by trained doctors. The examinations included measurement of the foot temperature sensation. For this procedure, a testing tool is placed onto the foot skin with its metal side (cooler) and polymer side (warmer); positivity was defined as inability to differentiate the two sides of the Tip Therm in either side of the foot. Besides examining the ABI and Achilles tendon reflex, a 10-g monofilament was gently bent for 1–2 s, and one side was pushed upon the arm skin to give the patient a reference for pressure sensation. The patient closed both eyes. For each foot, the pressure sensation was tested at the plantar surface of the great toe, the lateral side of the anterior sole, and medial side of the anterior sole. Positivity was defined as loss of pressure sensation in any tested sites of either foot. For vibration sensation, a 128 Hz tuning fork was placed onto the wrist or elbow to give the patient with a reference point for vibration or non-vibration. The patient closed both eyes. A vibrating tuning fork was then placed onto the dorsal surface of the metatarsal joint of the great toe. The patient was asked whether a vibration was felt. The test was repeated three times for both sides. Positivity was defined as two or three wrong answers for either side. Foot deformity included skeletal deformities of the foot (e.g., hallux valgus, toe deformity, Charcot foot) and callus formation. A history of hypertension, hyperlipidemia, chronic kidney disease, coronary heart disease and cerebrovascular disease were obtained from previous medical records.

### Diagnostic Criteria

Diagnostic criteria for DPN and PAD were based on the Guidelines for the Prevention and Control of Type 2 Diabetes in China (2017 Edition). Patients with clinical symptoms of neuropathy and one abnormal result from five examinations (ankle reflex, acupuncture pain sensation, vibration sensation, pressure sensation, and temperature sensation) were diagnosed with DPN. Patients without clinical symptoms of neuropathy and two abnormal results from the five examinations were also diagnosed with DPN. If the patient’s symptoms were in line with a diabetes diagnosis and they had an ABI of ≤0.9 at rest, then PAD was diagnosed. Foot deformity included skeletal deformities of the foot (hallux valgus, hammer toe, claw toe, mallet toe, and Charcot foot), and callus. Hallux valgus was defined as deformity of the great toe by abduction valgus and pronation associated with bone prominence on the inner edge of the metatarsal (bunion) (12). Hammer toe was defined as plantar flexion of the distal and middle interphalangeal joint in comparison to the proximal phalanx. Claw toe was defined as the dorsal flexion of the metatarsophalangeal joint associated with hammer toe (13). Mallet toe was defined as flexion of the distal phalanx over the middle phalanx due to a contracture at the distal interphalangeal joint (14). Charcot foot was defined as non-infectious destruction of bone and joint tissue including loss of foot arches, i.e., rocker bottom deformity (15). Callus was defined as a broad, diffuse area of hyperkeratosis with a relatively even thickness (16).

### Statistical Analyses

Statistical analyses were performed using SPSS software (version 19.0; SPSS Inc., Chicago, IL, USA). Normally distributed continuous variables were expressed as means ± standard deviations (SD). First, we compared characteristics such as age, sex, diabetes duration, SBP, BMI, HbA_1c_, and LDL-c levels between patients with and without DPN, PAD, or foot deformity. From a clinical point of view, we grouped age, duration of diabetes, BMI, SBP, HbA_1c_ and LDL-_C_ into categories. Categorical variables were compared using the Chi-squared test and reported as frequencies and proportions. Then we performed binary logistic regression analysis with the variables which show significant difference among the patients with and without DPN/PAD/foot deformity in order to identify potential risk factors for DPN, PAD, or foot deformity. All significant variables were added to the model simultaneously. Statistical significance was set at p<0.05.

## Results

### Baseline Characteristics

**Table 1** presents the baseline characteristics of the study sample. In total, 3,758 patients were included in the analyses; 39 patients had type 1 diabetes, and 3,719 had type 2 diabetes. There were 2,024 (53.9%) men and 1,734 (46.1%) women, the median age was 62.97 years, and the median diabetes duration was 11.33 years. The only different in clinical characteristics between men and women is that the average age of women was higher than that of men (64.76 ± 10.67 years vs 61.40 ± 11.67 years, p<0.001). Overall, 882 (23.5%) patients had DPN, 437 (11.6%) had PAD, and 1,117 (29.7%) had a foot deformity (including callus) (**Table 2**). In patients with foot deformity (n=1117), 566 were diagnosed with skeletal deformity of the foot, accounting for 15.1% of the total patient sample overall. In addition, 904 patients were diagnosed with callus, accounting for 24.1% of the total patient sample overall. The other diagnoses in patients with foot deformity were hallux valgus (n=503), toe deformity (n=95), and Charcot foot (n=3).

**Table 1:** Characteristics of the study participants.

Characteristics	Study participants (n = 3758)
Age (years)	62.97 ± 11.40
Women [n(%)]	1734 (46.1%)
Duration of diabetes (years)	11.33 ± 7.91
BMI (kg/m^2^)	25.53 ± 3.73
SBP (mmHg)	131.58 ± 15.66
HbA_1c_(%)	7.64 ± 1.74
LDL-c (mmol/L)	2.46 ± 0.81
Hypertension [n(%)]	2491(66.3%)
Hyperlipoidemia [n(%)]	1990 (53.0%)
Chronic kidney disease [n(%)]	149 (4.0%)
Coronary heart disease [n(%)]	1077 (28.7%)
Cerebrovascular disease [n(%)]	725 (19.3%)

BMI, body mass index; SBP, systolic blood pressure; HbA_1c_, glycosylated hemoglobin; LDL-c, low density lipoprotein.

**Table 2:** Prevalence of DPN, PAD and Foot Deformity in study participants.

Foot screening	n (%)
DPN	882 (23.5%)
PAD	437 (11.6%)
Foot Deformity	1117 (29.7%)

DPN, diabetic peripheral neuropathy; PAD, peripheral artery disease.

### DPN Risk Factors

We chose the variables which show significant difference among the patients with and without DPN (**Supplementary Table 1**). **Table 3** presents the logistic regression model outputs with DPN as the binary outcome. Age between 40-50, 50-60, 60-70, and ≥70 years, a 10-15 year and 10+ year diabetes duration, BMI <18.5 kg/m^2^ or ≥24 kg/m^2^, SBP ≥140 mm Hg, HbA_1c_ level ≥7%, chronic kidney disease, and cerebrovascular disease were risk factors for DPN. Among these risk factors, the OR for BMI<18.5 kg/m^2^ was the highest (**Table 3**). BMI levels appear to be non-linear in relation to DPN, so we further conducted smooth curve fitting(**Supplementary Figure 1**). Smooth curve fitting shows that there is a non-linear relationship between BMI and DPN. Further threshold effect analysis shows that the knee(K) is BMI=20.24kg/m^2^ (**Supplementary Table 2**). That is, if BMI is less than 20.24 kg/m^2^, BMI is negatively correlated with the prevalence of DPN. If BMI is greater than 20.24 kg/m^2^, BMI is positively correlated with the prevalence of DPN. LDL-c seems to be a protective factor for DPN. We further conducted subgroup analysis and interaction testing. As we can see in the **Supplementary Table 3**, there were no interaction between age, duration of diabetes, BMI, Hyperlipoidemia, Chronic kidney disease, Coronary heart disease, Cerebrovascular disease and LDL-c.

**Table 3:** Logistic regression analysis for factors associated with DPN.

	OR (95%CI)	P-value
Age (years)		
<40	Reference	
40-50	1.960 (1.020, 3.766)	0.043
50-60	2.537 (1.387, 4.642)	0.003
60-70	2.549 (1.402, 4.636)	0.002
≥70	2.627 (1.431, 4.822)	0.002
Duration of diabetes (years)		
<5	Reference	
5-10	1.000 (0.763, 1.311)	NS
10-15	1.629 (1.261, 2.106)	<0.001
≥15	1.898 (1.483, 2.429)	<0.001
BMI (kg/m^2^)		
<18.5	3.560 (1.932, 6.562)	<0.001
18.5-23.9	Reference	
24-27.9	1.238 (1.024, 1.496)	0.027
≥28	1.353 (1.083, 1.691)	0.008
SBP≥140mmHg	1.684 (1.416, 2.003)	<0.001
HbA_1c_ (%)		
<6.5	Reference	
6.5-6.9	0.930 (0.710, 1.217)	NS
7-7.9	1.396 (1.095, 1.779)	0.007
≥8.0	1.995 (1.590, 2.504)	<0.001
LDL-c(mmol/L)		
<1.8	Reference	
1.8-2.6	0.839 (0.680, 1.035)	NS
≥2.6	0.751 (0.606, 0.931)	0.009
Hyperlipoidemia	1.156 (0.981, 1.362)	NS
Chronic kidney disease	2.278 (1.597, 3.250)	<0.001
Coronary heart disease	0.859 (0.712, 1.035)	NS
Cerebrovascular disease	1.538 (1.267, 1.866)	<0.001

DPN, diabetic peripheral neuropathy; BMI, body mass index; SBP, systolic blood pressure; HbA_1c_, glycosylated hemoglobin; LDL-c, low density lipoprotein; NS, not significance.

### PAD Risk Factors

We chose the variables which show significant difference among the patients with and without PAD (**Supplementary Table 4**). **Table 4** presents the logistic regression model outputs with DBN as the binary outcome. A 15+ year diabetes duration, BMI <18.5 kg/m^2^, SBP ≥140 mm Hg, a HbA_1c_ level of ≥7%, chronic kidney disease, coronary heart disease, and cerebrovascular disease were significant risk factors for PAD. Interestingly, the same as DPN, the OR value of BMI<18.5 kg/m^2^ was the highest value. But BMI≥24 kg/m^2^ were not risk factors for PAD.

**Table 4:** Logistic regression analysis for factors associated with PAD.

	OR (95%CI)	P-value
Age (years)		
<40	Reference	
40-50	0.670 (0.308, 1.459)	NS
50-60	0.768 (0.392, 1.504)	NS
60-70	1.094 (0.571, 2.098)	NS
≥70	1.507 (0.777, 2.922)	NS
Duration of diabetes (years)		
<5	Reference	
5-10	0.879 (0.610, 1.267)	NS
10-15	1.236 (0.872, 1.752)	NS
≥15	1.565 (1.131, 2.167)	0.007
BMI (kg/m^2^)		
<18.5	2.464 (1.189, 5.104)	0.015
18.5-23.9	Reference	
24-27.9	1.169 (0.912, 1.497)	NS
≥28	1.188 (0.886, 1.592)	NS
SBP≥140mmHg	1.261 (1.001, 1.588)	0.049
HbA_1c_ (%)		
<6.5	Reference	
6.5-6.9	1.016 (0.708, 1.457)	NS
7-7.9	1.468 (1.063, 2.028)	0.020
≥8.0	1.877 (1.385, 2.542)	<0.001
LDL-c (mmol/L)		
<1.8	Reference	
1.8-2.6	0.868 (0.663, 1.137)	NS
≥2.6	0.850 (0.645, 1.119)	NS
Hypertension	0.875 (0.683, 1.121)	NS
Hyperlipoidemia	1.226 (0.984, 1.527)	NS
Chronic kidney disease	1.793 (1.183, 2.717)	0.006
Coronary heart disease	1.518 (1.205, 1.912)	<0.001
Cerebrovascular disease	1.511 (1.189, 1.921)	<0.001

PAD, peripheral artery disease; BMI, body mass index; SBP, systolic blood pressure; HbA_1c_, glycosylated hemoglobin; LDL-c, low density lipoprotein; NS, not significance.

### Foot Deformity Risk Factors

We chose the variables which show significant difference among the patients with and without foot skeletal deformity (**Supplementary Table 5**) or callus (**Supplementary Table 6**). **Tables 5** and **6** present the logistic regression model outputs for foot skeletal deformity and callus as the binary outcomes, respectively. Women, aged ≥40 years, SBP ≥140 mm Hg, and hyperlipidemia were risk factors for skeletal deformities of the foot. The risk factors for callus included women, SBP ≥140 mm Hg and hyperlipidemia. As we can see in **Table 5** and **Table 6**, age significantly affects the risk of foot skeletal deformity, while age was not a risk factor for callus.

**Table 5:** Logistic regression analysis for factors associated with foot skeletal deformity.

	OR (95%CI)	P-value
women	1.815 (1.501, 2.195)	<0.001
Age(years)		
<40	Reference	
40-50	2.981 (1.114, 7.977)	0.030
50-60	4.314 (1.711, 10.877)	0.002
60-70	4.980 (1.993, 12.446)	<0.001
≥70	5.855 (2.323, 14.755)	<0.001
Duration of diabetes(years)		
<5	Reference	
5-10	0.637 (0.480, 0.847)	0.002
10-15	0.792 (0.601, 1.045)	NS
≥15	0.838 (0.643, 1.092)	NS
BMI(kg/m^2^)		
<18.5	1.285 (0.612, 2.697)	NS
18.5-23.9	Reference	
24-27.9	0.876 (0.710, 1.079)	NS
≥28	0.713 (0.545, 0.933)	0.014
SBP≥140mmHg	1.556 (1.269, 1.907)	<0.001
HbA_1c_(%)		
<6.5	Reference	
6.5-6.9	0.878 (0.672, 1.147)	NS
7-7.9	0.903 (0.698, 1.167)	NS
≥8.0	0.521 (0.400, 0.679)	<0.001
LDL-c(mmol/L)		
<1.8	Reference	
1.8-2.6	0.696 (0.543, 0.893)	0.004
≥2.6	0.882 (0.690, 1.128)	NS
Hyperlipoidemia	1.313 (1.086, 1.586)	0.005
Coronary heart disease	0.580 (0.462, 0.728)	<0.001

BMI, body mass index; SBP, systolic blood pressure; HbA_1c_, glycosylated hemoglobin; LDL-c, low density lipoprotein; NS, not significance.

**Table 6:** Logistic regression analysis for factors associated with callus.

	OR (95%CI)	p-value
women	1.344 (1.146-1.577)	<0.001
Duration of diabetes (years)		
<5	Reference	
5-10	0.565 (0.443-0.720)	<0.001
10-15	0.705 (0.555-0.896)	0.004
≥15	0.935 (0.749-1.167)	NS
SBP≥140 mmHg	1.577 (1.321-1.884)	<0.001
HbA_1c_ (%)		
<6.5	Reference	
6.5-7.0	1.037 (0.822-1.308)	NS
7.0-8.0	1.009 (0.806-1.263)	NS
≥8.0	0.507 (0.404-0.636)	<0.001
Hypertension	0.673 (0.564-0.802)	<0.001
Hyperlipoidemia	1.662 (1.408-1.962)	<0.001
Coronary heart disease	0.828 (0.684-1.002)	NS
Cerebrovascular disease	0.865 (0.697-1.074)	NS

HbA_1c_, glycosylated hemoglobin; LDL-c, low density lipoprotein; NS, not significance.

## Discussion

DPN, PAD, and foot deformity are common in patients with diabetes, which brings physical and mental suffering to the patient and their family members, and negatively affects their quality of life. Understanding the prevalence and risk factors of DPN, PAD, and foot deformity are necessary to formulate a prevention strategy for diabetic foot in Chinese patients with diabetes. Therefore, we conducted a multicenter survey of 3,758 cases across 11 hospitals in Beijing to clarify the prevalence and risk factors of diabetic foot in a representative sample of Chinese patients with type I or type II diabetes.

DPN is an important risk factor in diabetic foot. The risk of diabetic foot ulcers is reported to be seven times greater in patients with DPN and sensory deficits than in patients without DPN and sensory deficits (17). A Chinese survey showed that 63.6% of diabetic foot ulcers were caused by DPN (18). DPN was defined as a major risk factor for diabetic foot by the IWGDF (1). In this study, we found that age ≥40 years, a ≥10-year duration of diabetes, underweight (BMI<18.5 kg/m^2^) or overweight and obesity (BMI >24 kg/m^2^), SBP ≥140 mm Hg, a HbA_1c_ level of ≥7%, chronic kidney disease, and cerebrovascular disease were all risk factors of DPN. Consistent with our findings, The Action to Control Cardiovascular Risk in Diabetes (ACCORD) trial suggested that lowering blood sugar slowed the occurrence of DPN (19). Further, a meta-analysis identified diabetes duration, age, glycosylated hemoglobin, and diabetic retinopathy as risk factors for DBN (20). The results of our study showed that chronic kidney disease and underweight also increased the risk of DPN. Chronic kidney disease, especially renal insufficiency, can lead to systemic vascular diseases, including microvascular diseases (21, 22). Microvascular diseases lead to a decrease in peripheral nerve blood flow, causing DPN. Underweight is often accompanied by nutrient deficiency, which causes a decrease in neurotrophic factors and leads to DPN (23). Another interesting finding is that the DPN risk was lower in patients with an LDL-c level of ≥2.6 mmol/L than in patients with an LDL-c of <1.8 mmol/L. However, hyperlipidemia did not reduce the risk of DPN. We suspect that lipid-lowering drugs may influence neuropathy, but further research is needed to confirm this hypothesis.

In this study, we found that a diabetes duration of ≥15 years, underweight (BMI<18.5 kg/m^2^), SBP of ≥140 mm Hg, HbA_1c_ level of ≥7%, chronic kidney disease, and cardiovascular and cerebrovascular diseases were risk factors for PAD. Previous research has shown that age, hypertension, dyslipidemia, and smoking are PAD risk factors (24). Furthermore, patients aged >50 years with cardiovascular and cerebrovascular diseases, dyslipidemia, hypertension, smoking, or diabetes for more than 5 years are recommended to undergo screening for PAD every year for early detection (25, 26). Coronary heart disease or cerebrovascular disease indicates atherosclerosis in the blood vessels of the heart or brain. PAD is not a specific complication of diabetes, but one of the most common manifestations of atherosclerosis in the lower extremities. Therefore, it is not difficult to understand that coronary heart disease or cerebrovascular disease were risk factors for PAD. Patients with chronic kidney disease are at increased risk of atherosclerosis (27), this could explain chronic kidney disease as a risk factor for PAD.

According to the results of this study, the prevalence of foot deformity was higher than DPN and PAD. However, IWGDF pays more attention to DPN than PAD and foot deformity. Therefore, foot deformities may be missed in many patients. In this study, we found both risk and protective factors for foot deformity in patients with diabetes. The risk of skeletal deformity of the foot increased with age, and was higher in women than in men, which could be attributed to walking and inappropriate shoes. However, coronary heart disease, obesity, and an elevated HbA_1c_ level may be protective factors for skeletal foot deformity, potentially related to changes in activity habits. Foot deformity leads to a change in plantar pressure, increasing the incidence of foot ulcers (28). In the non-diabetic population, foot deformity is related to shoes or environmental factors (29). Angina pectoris and cardiac insufficiency caused by coronary heart disease lead to decreased activity tolerance, reducing the risk of skeletal foot deformity. Obesity and elevated blood sugar are also related to a sedentary lifestyle and inactivity. Thus, the protective effect of obesity and an elevated HbA_1c_ level on skeletal foot deformities may also come from reduced activity. This kind of obesity paradox was also found in foot ulceration risk (30) and lower-extremity amputation risk (31), but lacked a valid explanation. In our study, lipids affected skeletal foot deformities in two ways. On one hand, hyperlipidemia increased the risk of skeletal deformity; on the other hand, an LDL-c level of ≤1.8 mmol/L did not reduce the risk of skeletal foot deformities, perhaps owing to cholesterol or statin use. Further research is needed in order to confirm this.

Callus increase plantar pressure, plantar pressure duration (32, 33), and the risk of diabetic foot ulcers. Callus are caused by excessive pressure and friction of the skin of the foot, often related to shoes. Similar to skeletal foot deformities, women had a greater risk of callus, likely resulting from shoe habits. An increased HbA_1c_ level may be related to insufficient activity. Compared to a diabetes duration of fewer than 5 years, the risk of callus decreased when the diabetes duration was 5–15 years but was similar when the duration was more than 15 years. Thus, diabetes duration is a protective factor for callus. A history of hypertension reduced the risk of callus, but a higher SBP increased the risk, suggesting that some antihypertensive drugs may have a protective effect regarding callus, but further research is required.

There are certain limitations to our study. First, nerve conduction studies were not performed, potentially leading to missed diagnoses. Patients with PAD may have a non-compressible ABI due to medial calcinosis, which would be undetected using only the criterion of lower than 0.90. We also did not examine smoking history, which is a known risk factor for PAD. Second, our study focused on patients visiting clinics and did not cover a large number of patients who were unaware of their condition or unwilling to be treated. The cross-sectional nature of this study limits our ability to infer any causal effects. A prospective follow-up study is required to evaluate the associations between exposures and outcomes.

In conclusion, despite the high prevalence of DPN, PAD, and foot deformity in Chinese patients with diabetes, these complications were underdiagnosed and undertreated. Strengthening the management of risk factors for diabetic foot might improve the quality of life and lifespan of patients with diabetes and reducing their medical burden.

## Data Availability Statement

The original contributions presented in the study are included in the article/**Supplementary Material**. Further inquiries can be directed to the corresponding author.

## Ethics Statement

The studies involving human participants were reviewed and approved by Biomedical Research Ethics Committee, Peking University First Hospital. The patients/participants provided their written informed consent to participate in this study.

## Author Contributions

JYL, XY, and JL designed the study, and conducted the literature research. JYL and XY carried out the data interpretation, and drafted the manuscript. YS, DZ, and HL carried out data acquisition. GY, XQ, JZ, BW, and XG contributed to interpretation of the data and significantly improved the manuscript. All authors read and approved the final manuscript.

## Funding

This study was supported by Beijing Municipal Science and Technology Commission (grant number D141107005314003 and Z181100001718121).

## Conflict of Interest

The authors declare that the research was conducted in the absence of any commercial or financial relationships that could be construed as a potential conflict of interest.

## Publisher’s Note

All claims expressed in this article are solely those of the authors and do not necessarily represent those of their affiliated organizations, or those of the publisher, the editors and the reviewers. Any product that may be evaluated in this article, or claim that may be made by its manufacturer, is not guaranteed or endorsed by the publisher.
