# Supplementary material for: Risk Factors for Diabetic Peripheral Neuropathy, Peripheral Artery Disease, and Foot Deformity Among the Population With Diabetes in Beijing, China: A Multicenter, Cross-Sectional Study
Source: Front Endocrinol (Lausanne). 2022 Jun 6;13:824215. doi: 10.3389/fendo.2022.824215 (PMC9207340; doi:10.3389/fendo.2022.824215)
Supplement: Supplementary file 1 [file DataSheet_1.docx]

Supplementary Material

# Supplementary Tables

Supplementary Table 1. Characteristics among patients with and without DPN

|  | without DPN  (n=2876) | DPN  (n=882) | *χ*^2^ | P-value |
| --- | --- | --- | --- | --- |
| Age, n (%) |  |  | 24.070 | <0.001 |
| < 40 years | 127(4.4%) | 14(1.6%) |  |  |
| 40 - 50 years | 232(8.1%) | 53(6.0%) |  |  |
| 50 - 60 years | 645(22.4%) | 199(22.6%) |  |  |
| 60 - 70 years | 1146(39.8%) | 351(39.8%) |  |  |
| ≥ 70 years | 726(25.2%) | 265(30.0%) |  |  |
| Duration of diabetes, n (%) |  |  | 108.714 | <0.001 |
| < 5 years | 658(22.9%) | 123(13.9%) |  |  |
| 5 - 10 years | 792(27.5%) | 154(17.5%) |  |  |
| 10 - 15 years | 647(22.5%) | 231(26.2%) |  |  |
| ≥ 15 years | 779(27.1%) | 374(42.4%) |  |  |
| Women, n (%) | 1345(46.8%) | 389(44.1%) | 1.925 | 0.165 |
| Hypertension, n (%) | 1896(65.9%) | 595(67.5%) | 0.712 | 0.399 |
| Hyperlipoidemia, n (%) | 1472(51.2%) | 518(58.7%) | 15.436 | <0.001 |
| Chronic kidney disease, n (%) | 80(2.8%) | 69(7.8%) | 45.057 | <0.001 |
| Coronary heart disease, n (%) | 807(28.1%) | 270(30.6%) | 2.151 | 0.142 |
| Cerebrovascular disease, n (%) | 481(16.7%) | 244(27.7%) | 51.883 | <0.001 |
| BMI, n (%) |  |  | 18.784 | <0.001 |
| < 18.5 kg/m^2^ | 17(0.6%) | 10(1.1%) |  |  |
| 18.5 - 23.9 kg/m^2^ | 1053(36.6%) | 263(29.8%) |  |  |
| 24 - 27.9 kg/m^2^ | 1208(42.0%) | 387(43.9%) |  |  |
| ≥ 28 kg/m^2^ | 598(20.8%) | 222(25.2%) |  |  |
| SBP ≥ 140mmHg, n (%) | 648(22.5%) | 327(37.1%) | 74.011 | <0.001 |
| HbA_1c_, n (%) |  |  | 89.146 | <0.001 |
| < 6.5% | 764(26.5%) | 152(17.2%) |  |  |
| 6.5 - 7.0% | 623(21.7%) | 126(14.3%) |  |  |
| 7.0 - 8.0% | 684(23.8%) | 226(25.6%) |  |  |
| ≥ 8.0% | 805(28.0%) | 378(42.9%) |  |  |
| LDL-c, n (%) |  |  | 15.238 | <0.001 |
| < 1.8 mmol/L | 561(19.5%) | 225(25.5%) |  |  |
| 1.8 - 2.6 mmol/L | 1153(40.1%) | 339(38.4%) |  |  |
| ≥ 2.6 mmol/L | 1162(40.4%) | 318(36.1%) |  |  |

Abbreviations: DPN, diabetic peripheral neuropathy; BMI, body mass index; SBP, systolic blood pressure; HbA_1c_, glycosylated hemoglobin; LDL-c, low density lipoprotein

Supplementary Table 2. Threshold effect analysis of DPN and BMI

|  | OR (95%CI) | P-value |
| --- | --- | --- |
| < K | 0.75 (0.62, 0.90) | 0.002 |
| > K | 1.04 (1.02, 1.06) | <0.001 |

Abbreviations: K, knee; DPN, diabetic peripheral neuropathy; BMI, body mass index.

Supplementary Table 3. Subgroup analysis and interaction testing for DPN between LDL-c and other factors

|  | LDL-c(mmol/L) | | | | |  |
| --- | --- | --- | --- | --- | --- | --- |
|  | <1.8 | >=1.8, <2.6 | | >=2.6 | |  |
|  |  | OR | P-value | OR | P-value | P interaction |
| Age |  |  |  |  |  |  |
| <40 years | 1 | 0.280 (0.020, 3.882) | 0.343 | 0.685 (0.062, 7.612) | 0.758 | 0.388 |
| 40 - 50 years | 1 | 0.563 (0.247, 1.284) | 0.172 | 0.465 (0.200, 1.078) | 0.074 |  |
| 50 - 60 years | 1 | 0.822 (0.506, 1.334) | 0.427 | 0.919 (0.575, 1.469) | 0.724 |  |
| 60 - 70 years | 1 | 0.796 (0.569, 1.112) | 0.180 | 0.781 (0.555, 1.100) | 0.157 |  |
| ≥ 70 years | 1 | 1.042 (0.711, 1.527) | 0.832 | 0.648 (0.430, 0.976) | 0.038 |  |
| Duration of diabetes |  |  |  |  |  |  |
| < 5 years | 1 | 0.978 (0.565, 1.693) | 0.937 | 0.780 (0.457, 1.331) | 0.363 | 0.662 |
| 5 - 10 years | 1 | 0.666 (0.399, 1.111) | 0.119 | 0.694 (0.418, 1.153) | 0.159 |  |
| 10 - 15 years | 1 | 0.686 (0.450, 1.045) | 0.079 | 0.567 (0.365, 0.881) | 0.012 |  |
| ≥ 15 years | 1 | 0.984 (0.708, 1.366) | 0.922 | 0.892 (0.634, 1.255) | 0.512 |  |
| BMI |  |  |  |  |  |  |
| < 18.5 kg/m^2^ | 1 | 0.717 (0.486, 1.056) | 0.092 | 0.681 (0.459, 1.009) | 0.055 | 0.642 |
| 18.5 - 23.9 kg/m^2^ | 1 | 0.636 (0.051, 7.994) | 0.726 | 2.606 (0.162, 41.913) | 0.499 |  |
| 24 - 27.9 kg/m^2^ | 1 | 0.885 (0.643, 1.219) | 0.454 | 0.695 (0.500, 0.966) | 0.030 |  |
| ≥ 28 kg/m^2^ | 1 | 0.938 (0.612, 1.437) | 0.767 | 0.910 (0.590, 1.404) | 0.670 |  |
| Hyperlipoidemia |  |  |  |  |  |  |
| NO | 1 | 0.836 (0.600, 1.164) | 0.288 | 0.752 (0.537, 1.052) | 0.096 | 0.991 |
| YES | 1 | 0.840 (0.638, 1.105) | 0.213 | 0.755 (0.570, 1.000) | 0.050 |  |
| Chronic kidney disease |  |  |  |  |  |  |
| NO | 1 | 0.797 (0.641, 0.990) | 0.040 | 0.726 (0.583, 0.905) | 0.004 | 0.215 |
| YES | 1 | 1.686 (0.703, 4.049) | 0.242 | 1.363 (0.507, 3.668) | 0.540 |  |
| Coronary heart disease |  |  |  |  |  |  |
| NO | 1 | 0.828 (0.639, 1.074) | 0.155 | 0.753 (0.581, 0.977) | 0.033 | 0.975 |
| YES | 1 | 0.902 (0.624, 1.305) | 0.586 | 0.819 (0.551, 1.218) | 0.323 |  |
| Cerebrovascular disease |  |  |  |  |  |  |
| NO | 1 | 0.858 (0.670, 1.098) | 0.224 | 0.778 (0.605, 1.001) | 0.051 | 0.895 |
| YES | 1 | 0.734 (0.484, 1.113) | 0.146 | 0.649 (0.424, 0.995) | 0.047 |  |

Abbreviations: DPN, diabetic peripheral neuropathy; LDL-c, low density lipoprotein; BMI, body mass index.

Supplementary Table 4. Characteristics among patients with and without PAD

|  | without PAD  (n=3321) | PAD  (n=437) | *χ*^2^ | P-value |
| --- | --- | --- | --- | --- |
| Age, n (%) |  |  | 46.082 | <0.001 |
| < 40 years | 129(3.9%) | 12(2.7%) |  |  |
| 40 - 50 years | 266(8.0%) | 19(4.3%) |  |  |
| 50 - 60 years | 776(23.4%) | 68(15.6%) |  |  |
| 60 - 70 years | 1327(40.0%) | 170(38.9%) |  |  |
| ≥ 70 years | 823(24.8%) | 168(38.4%) |  |  |
| Duration of diabetes, n (%) |  |  | 53.261 | <0.001 |
| < 5 years | 719(21.&%) | 62(14.2%) |  |  |
| 5 - 10 years | 868(26.1%) | 78(17.8%) |  |  |
| 10 - 15 years | 777(23.4%) | 101(23.1%) |  |  |
| ≥ 15 years | 957(28.8%) | 196(44.9%) |  |  |
| Women, n (%) | 1544(46.5%) | 190(43.5%) | 1.411 | 0.235 |
| Hypertension, n (%) | 2181(65.7%) | 310(70.9%) | 4.791 | 0.029 |
| Hyperlipoidemia, n (%) | 1726(52.0%) | 264(60.4%) | 11.041 | 0.001 |
| Chronic kidney disease, n (%) | 111(3.3%) | 38(8.7%) | 29.065 | <0.001 |
| Coronary heart disease, n (%) | 894(26.9%) | 183(41.9%) | 42.255 | <0.001 |
| Cerebrovascular disease, n (%) | 590(17.8%) | 135(30.9%) | 42.737 | <0.001 |
| BMI, n (%) |  |  | 7.313 | 0.063 |
| < 18.5 kg/m^2^ | 20(0.6%) | 6(1.4%) |  |  |
| 18.5 - 23.9 kg/m^2^ | 1181(35.6%) | 135(30.9%) |  |  |
| 24 - 27.9 kg/m^2^ | 1406(42.3%) | 189(43.2%) |  |  |
| ≥ 28 kg/m^2^ | 714((21.5%) | 107(24.5%) |  |  |
| SBP ≥ 140mmHg, n (%) | 829(25.0%) | 146(33.4%) | 14.257 | <0.001 |
| HbA_1c_, n (%) |  |  | 33.014 | <0.001 |
| < 6.5% | 840(25.3%) | 74(16.9%) |  |  |
| 6.5 - 7.0% | 681(20.5%) | 65(14.9%) |  |  |
| 7.0 - 8.0% | 797(24.0%) | 117(26.8%) |  |  |
| ≥ 8.0% | 1003(30.2%) | 181(41.4%) |  |  |
| LDL-c, n (%) |  |  | 10.430 | 0.005 |
| < 1.8 mmol/L | 668(20.1%) | 117(26.8%) |  |  |
| 1.8 - 2.6 mmol/L | 1325(39.9%) | 166(38.0%) |  |  |
| ≥ 2.6 mmol/L | 1328(40.0%) | 154(35.2%) |  |  |

Abbreviations: PAD, peripheral artery disease; BMI, body mass index; SBP, systolic blood pressure; HbA_1c_, glycosylated hemoglobin; LDL-c, low density lipoprotein

Supplementary Table 5. Characteristics among patients with and without foot skeletal deformity

|  | without foot skeletal deformity (n=3192) | foot skeletal deformity (n=566) | *χ*^2^ | P-value |
| --- | --- | --- | --- | --- |
| Age, n (%) |  |  | 32.131 | <0.001 |
| < 40 years | 136(4.3%) | 5(0.9%) |  |  |
| 40 - 50 years | 258(8.1%) | 27(4.8%) |  |  |
| 50 - 60 years | 731(22.9%) | 113(20.0%) |  |  |
| 60 - 70 years | 1256(39.3%) | 241(42.6%) |  |  |
| ≥ 70 years | 811(25.4%) | 180(31.8%) |  |  |
| Duration of diabetes, n (%) |  |  | 10.139 | 0.017 |
| < 5 years | 652(20.4%) | 129(22.8%) |  |  |
| 5 - 10 years | 833(26.1%) | 113(20.0%) |  |  |
| 10 - 15 years | 743(23.3%) | 135(23.9%) |  |  |
| ≥ 15 years | 964(30.2%) | 189(33.4%) |  |  |
| Women, n (%) | 1401(43.9%) | 333(58.8%) | 43.197 | <0.001 |
| Hypertension, n (%) | 2127(66.6%) | 364(64.3%) | 1.162 | 0.281 |
| Hyperlipoidemia, n (%) | 1660(52.0%) | 330(58.3%) | 7.656 | 0.006 |
| Chronic kidney disease, n (%) | 126(3.9%) | 23(4.1%) | 0.017 | 0.896 |
| Coronary heart disease, n (%) | 952(29.8%) | 125(22.1%) | 14.086 | <0.001 |
| Cerebrovascular disease, n (%) | 628(19.7%) | 97(17.1%) | 1.986 | 0.159 |
| BMI, n (%) |  |  | 10.524 | 0.015 |
| < 18.5 kg/m^2^ | 22(0.6%) | 4(0.7%) |  |  |
| 18.5 - 23.9 kg/m^2^ | 1091(34.2%) | 226(39.9%) |  |  |
| 24 - 27.9 kg/m^2^ | 1357(42.5%) | 237(41.9%) |  |  |
| ≥ 28 kg/m^2^ | 722(22.6%) | 99(17.5%) |  |  |
| SBP ≥ 140mmHg, n (%) | 787(24.7%) | 188(33.2%) | 18.482 | <0.001 |
| HbA_1c_, n (%) |  |  | 24.208 | <0.001 |
| < 6.5% | 753(23.6%) | 163(28.7%) |  |  |
| 6.5 - 7.0% | 622(19.5%) | 123(21.7%) |  |  |
| 7.0 - 8.0% | 760(23.8%) | 151(26.7%) |  |  |
| ≥ 8.0% | 1057(33.1%) | 129(22.8%) |  |  |
| LDL-c, n (%) |  |  | 6.034 | 0.049 |
| < 1.8 mmol/L | 651(20.4%) | 135(23.9%) |  |  |
| 1.8 - 2.6 mmol/L | 1290(40.4%) | 200(35.3%) |  |  |
| ≥ 2.6 mmol/L | 1251(39.2%) | 231(40.8%) |  |  |

Abbreviations: BMI, body mass index; SBP, systolic blood pressure; HbA_1c_, glycosylated hemoglobin; LDL-c, low density lipoprotein

Supplementary Table 6. Characteristics among patients with and without callus

|  | without callus  (n=2854) | callus  (n=904) | *χ*^2^ | P-value |
| --- | --- | --- | --- | --- |
| Age, n (%) |  |  | 7.713 | 0.103 |
| < 40 years | 110(3.9%) | 31(3.4%) |  |  |
| 40 - 50 years | 212(7.4%) | 73(8.1%) |  |  |
| 50 - 60 years | 645(22.6%) | 199(22.0%) |  |  |
| 60 - 70 years | 1108(38.8%) | 389(43.0%) |  |  |
| ≥ 70 years | 779(27.3%) | 212(23.5%) |  |  |
| Duration of diabetes, n (%) |  |  | 29.850 | <0.001 |
| < 5 years | 564(19.8%) | 217(24.0%) |  |  |
| 5 - 10 years | 772(27.0%) | 174(19.2%) |  |  |
| 10 - 15 years | 679(23.8%) | 199(22.0%) |  |  |
| ≥ 15 years | 839(29.4%) | 314(34.7%) |  |  |
| Women, n (%) | 1290(45.2%) | 444(49.1%) | 4.235 | 0.040 |
| Hypertension, n (%) | 1936(67.8%) | 555(61.4%) | 12.744 | <0.001 |
| Hyperlipoidemia, n (%) | 1436(50.3%) | 554(61.3%) | 33.150 | <0.001 |
| Chronic kidney disease, n (%) | 107(3.7%) | 42(4.6%) | 1.450 | 0.228 |
| Coronary heart disease, n (%) | 859(30.1%) | 218(24.1%) | 12.020 | 0.001 |
| Cerebrovascular disease, n (%) | 581(20.4%) | 144(15.9%) | 8.646 | 0.003 |
| BMI, n (%) |  |  | 2.621 | 0.454 |
| < 18.5 kg/m^2^ | 23(0.8%) | 3(0.3%) |  |  |
| 18.5 - 23.9 kg/m^2^ | 1004(35.2%) | 313(34.6%) |  |  |
| 24 - 27.9 kg/m^2^ | 1210(42.4%) | 384(42.5%) |  |  |
| ≥ 28 kg/m^2^ | 617(21.6%) | 204(22.6%) |  |  |
| SBP ≥ 140mmHg, n (%) | 679(23.8%) | 296(32.7%) | 29.249 | <0.001 |
| HbA_1c_, n (%) |  |  | 40.025 | <0.001 |
| < 6.5% | 691(24.2%) | 225(24.9%) |  |  |
| 6.5 - 7.0% | 539(18.9%) | 208(23.0%) |  |  |
| 7.0 - 8.0% | 651(22.8%) | 259(28.7%) |  |  |
| ≥ 8.0% | 973(34.1%) | 212(23.4%) |  |  |
| LDL-c, n (%) |  |  | 2.549 | 0.280 |
| < 1.8 mmol/L | 579(20.3%) | 205(22.7%) |  |  |
| 1.8 - 2.6 mmol/L | 1136(39.8%) | 357(39.5%) |  |  |
| ≥ 2.6 mmol/L | 1139(39.9%) | 342(37.8%) |  |  |

Abbreviations: BMI, body mass index; SBP, systolic blood pressure; HbA_1c_, glycosylated hemoglobin; LDL-c, low density lipoprotein

# Supplementary Figures

**Supplementary Figure 1.** Smooth curve fitting between BMI and DPN.

**
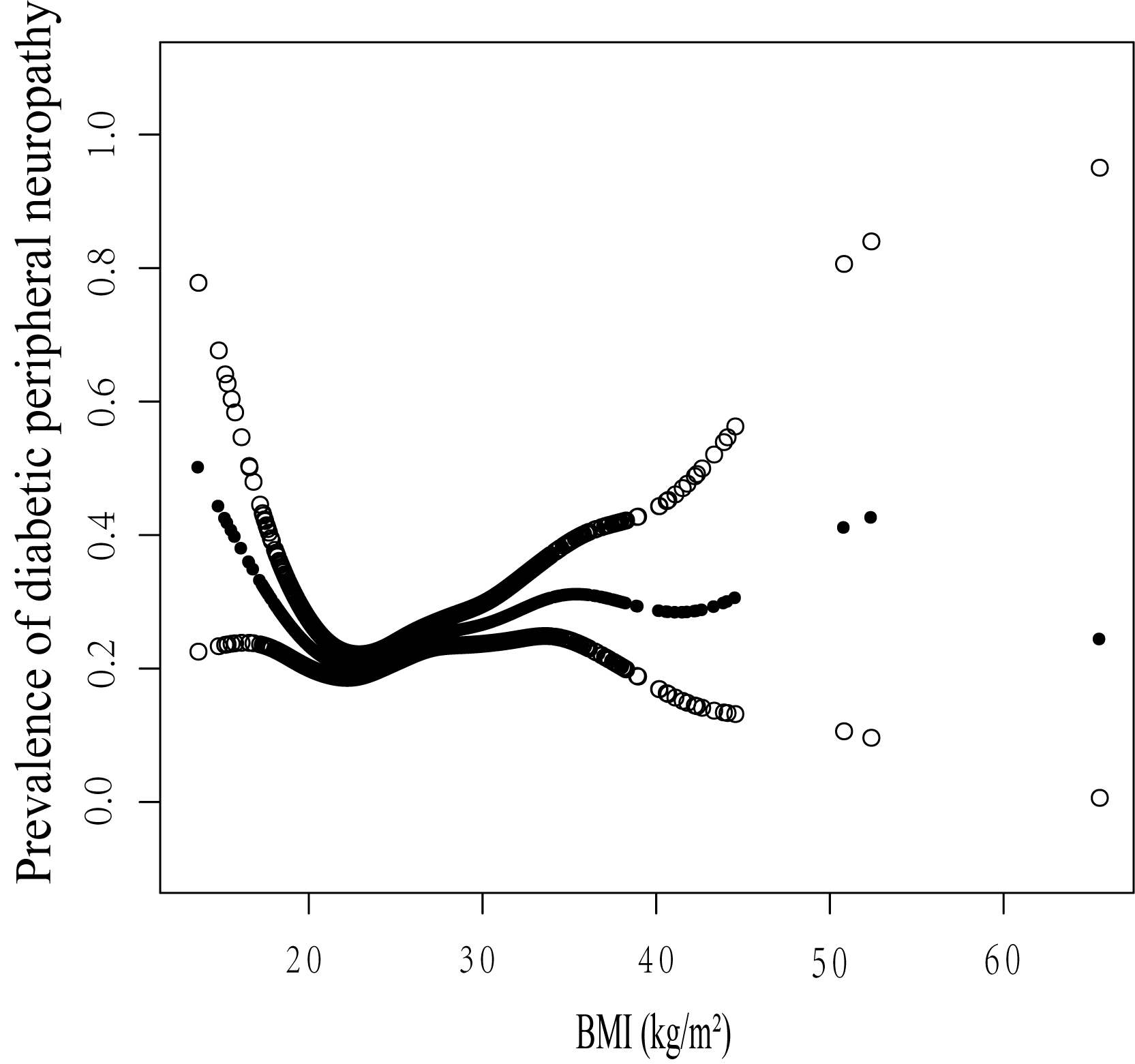
**
